# Spred2 Regulates High Fat Diet-Induced Adipose Tissue Inflammation, and Metabolic Abnormalities in Mice

**DOI:** 10.3389/fimmu.2019.00017

**Published:** 2019-01-22

**Authors:** Takahiro Ohkura, Teizo Yoshimura, Masayoshi Fujisawa, Toshiaki Ohara, Rie Marutani, Kaya Usami, Akihiro Matsukawa

**Affiliations:** Department of Pathology and Experimental Medicine, Okayama University Graduate School of Medicine, Dentistry and Pharmaceutical Sciences, Okayama, Japan

**Keywords:** Spred2, Ras/Raf/ERK/MAPK, obesity, macrophage, adipocyte, inflammation

## Abstract

Chronic low-grade inflammation in visceral adipose tissues triggers the development of obesity-related insulin resistance, leading to the metabolic syndrome, a serious health condition with higher risk of cardiovascular disease, diabetes, and stroke. In the present study, we investigated whether Sprouty-related EVH1-domain-containing protein 2 (Spred2), a negative regulator of the Ras/Raf/ERK/MAPK pathway, plays a role in the development of high fat diet (HFD)-induced obesity, adipose tissue inflammation, metabolic abnormalities, and insulin resistance. Spred2 knockout (KO) mice, fed with HFD, exhibited an augmented body weight gain, which was associated with enhanced adipocyte hypertrophy in mesenteric white adipose tissue (mWAT) and deteriorated dyslipidemia, compared with wild-type (WT) controls. The number of infiltrating macrophages with a M1 phenotype, and the crown-like structures, composed of macrophages surrounding dead or dying adipocytes, were more abundant in Spred2 KO-mWAT compared to in WT-mWAT. Exacerbated adipose tissue inflammation in Spred2 KO mice led to aggravated insulin resistance and fatty liver disease. To analyze the mechanism(s) that caused adipose tissue inflammation, cytokine response in mWAT was investigated. Stromal vascular fraction that contained macrophages from Spred2 KO-mWAT showed elevated levels of tumor necrosis factor α (TNFα) and monocyte chemoattractant protein-1 (MCP-1/CCL2) compared with those from WT-mWAT. Upon stimulation with palmitate acid (PA), bone marrow-derived macrophages (BMDMs) derived from Spred2 KO mice secreted higher levels of TNFα and MCP-1 than those from WT mice with enhanced ERK activation. U0126, a MEK inhibitor, reduced the PA-induced cytokine response. Taken together, these results suggested that Spred2, in macrophages, negatively regulates high fat diet-induced obesity, adipose tissue inflammation, metabolic abnormalities, and insulin resistance by inhibiting the ERK/MAPK pathway. Thus, Spred2 represents a potential therapeutic tool for the prevention of insulin resistance and resultant metabolic syndrome.

## Introduction

Metabolic syndrome is characterized by abdominal obesity, dyslipidemia, elevated blood pressure, and impaired glucose tolerance. Patients suffering from metabolic syndrome have twice the risk of mortality due to cardiovascular disease-associated events, such as stroke or myocardial infarction, compared with normal individuals (1). The International Diabetes Federation (IDF) estimates that one-quarter of the world's adult population has metabolic syndrome (2). Thus, metabolic syndrome is a serious threat to human health.

The principal determinant and core feature of metabolic syndrome is abdominal obesity (3); although, insulin resistance has also been recognized as the integral feature of this syndrome (4). Obesity is associated with a state of chronic, low-grade inflammation in the adipose tissue, which involves adipocyte hypertrophy, macrophage infiltration, and adipocyte-macrophage interaction (5). Saturated fatty acid (SFA) from hypertrophied adipocytes appears to activate toll-like receptor (TLR) 4 signaling pathway in adipocytes and macrophages, resulting in the production of proinflammatory cytokine, tumor necrosis factor α (TNFα) (6). The absence of TNFα resulted in significantly enhanced insulin sensitivity in both diet-induced and leptin-deficient (ob/ob) mouse models of obesity (7). In addition, the chemokine monocyte chemoattractant protein-1 (MCP-1/CCL2), and its receptor C-C motif chemokine receptor 2 (CCR2) play a critical role in macrophage infiltration and insulin resistance associated with obesity (8, 9).

TNFα activates multiple intracellular signaling pathways, including the nuclear factor (NF)-κB and the mitogen-activated protein kinase (MAPK) pathways (10). MCP-1 is also known to activate MAPK pathway (11). The MAPK family is composed of the extracellular signal-regulated kinase (ERK)-1/2, c-Jun amino-terminal kinases (JNKs), and p38 (12). The ERK/MAPK pathway is crucial in the initiation of the preadipocytes differentiation (13). Mice lacking ERK1 were resistant to high fat diet (HFD)-induced obesity and exhibited reduced insulin resistance (14). In addition, ERK signaling is essential for macrophage development (15), and stimulation in response to not only lipopolysaccharide (LPS) (16–18) but also free fatty acid (FFA) (19) via TLR4 (6, 20). These previous findings suggested that ERK/MAPK is involved in adipose tissue inflammation and remodeling.

Sprouty-related EVH1-domain-containing protein 2 (Spred2) is a member of the Spred protein family and inhibits the Ras/Raf/ERK/MAPK pathway (21). Spred2 ubiquitously expressed in various tissues including digestive tract, lung, kidney, and liver (22). As the ERK/MAPK pathway is involved in several cellular processes in adipocytes or macrophages, dysregulation of ERK/MAPK signaling could affect diet-induced obesity and obesity-induced adipose tissue inflammation and metabolic abnormalities. We previously reported that Spred2-deficiency in mice exacerbated acetaminophen-induced hepatotoxicity (23), LPS-induced acute lung inflammation (17), D-galactosamine/LPS-induced acute liver injury (18), and polymicrobial septic peritonitis (24). In the present study, we demonstrate, for the first time, that Spred2 plays an important role in regulating the development of diet-induced obesity, obesity-induced adipose tissue inflammation, and metabolic abnormalities, including fatty liver disease.

## Materials and Methods

### Mice

Spred2 KO mice onto a C57BL/6J background were kindly provided by Dr. Akihiko Yoshimura (25, 26). The lack of Spred2 expression in the mesenteric white adipose tissue (mWAT) and the liver of Spred2 KO mice was confirmed by TaqMan real-time quantitative polymerase chain reaction (RT-qPCR) (data not shown). C57BL/6J mice were used as wild-type (WT) control. WT and Spred2 KO mice were bred and maintained under continuous 12-h light and 12-h dark cycle under specific pathogen-free conditions at the Department of Animal Resources, Okayama University, Okayama, Japan. Male mice were used in this study. All animal protocols were approved by the Animal Care and Use Committee of the Okayama University, and all experiments were performed in accordance with relevant guidelines and regulations.

### Diet-Induced Obesity Model

Mice were weaned from parents at 4 weeks of age and given free access to water and either the standard diet (SD: 359 kcal/100 g; 12.8% energy as fat; MF, Oriental Yeast Co. Ltd., Tokyo, Japan), or high fat diet (HFD: 506 kcal/100 g; 60.0% energy as fat; HFD-60, Oriental Yeast Co. Ltd.). At different time intervals, mice were anesthetized, blood was collected by cardiac puncture, and mWAT and liver were excised. A part of the tissue was snap-frozen in liquid nitrogen and stored at −80°C for subsequent analyses. Another part of the tissue was fixed in 10% formalin, embedded in paraffin, and thin sections were prepared and histologically examined. Alternatively, livers were frozen in O.C.T compound and cryostat sections were stained by oil red O as per the standard techniques. In another set of experiments, a whole mWAT was resected and used for isolation of adipocytes and stromal vascular fraction as described below.

### Micro-Computed Tomography (Micro-CT) Image Acquisition

Mice (20 weeks of age) were anesthetized with isoflurane (Mylan Inc., Canonsburg, PA, USA) and scanned by micro-CT (LaTheta LCT-200; Hitachi Aloka Medical, Ltd., Tokyo, Japan). The abdominal adiposity was assessed using image-analyzing software (LaTheta v1.20).

### Blood Analysis

Serum total cholesterol, triglyceride, and blood glucose concentrations were measured using standard protocols. The serum insulin concentrations were measured using an insulin assay kit (Morinaga Institute of Biological Science Inc., Tokyo, Japan). The serum FFA concentrations were measured using a LabAssay NEFA kit (Wako Pure Chemical Industries Inc., Osaka, Japan).

### Glucose and Insulin Tolerance Tests

After 16 weeks of feeding (20 weeks of age), glucose and insulin tolerance tests were conducted by intraperitoneally injecting glucose (1 g/kg body weight, Sigma-Aldrich, St. Louis, MO, USA) and insulin (1 U/kg body weight, Eli Lilly Japan K.K., Hyogo, Japan), respectively, as previously described (27). Blood was sampled from the tail vein of mice, and blood glucose level was evaluated using a blood glucose test meter (Lab Gluco: ForaCare Suisse AG, St. Gallen, Switzerland).

### Isolation of Adipocytes and Stromal Vascular Fraction (SVF)

mWAT was minced and digested using type II collagenase (Sigma-Aldrich) dissolved in PBS at 37°C for 20 min on an orbital shaker. The digested tissues were centrifuged at 500 × g for 10 min. The pelleted cells represented the SVF, and the floating cells represented the adipocyte fraction (28).

### Isolation and Culture of Bone Marrow-Derived Macrophage (BMDM)

Bone marrow (BM) was harvested from the femur and tibia of WT or Spred2 KO mice. For generation of BMDMs, BM cells were cultured in RPMI-1640 medium, supplemented with 10% FBS and antibiotics, in the presence of M-CSF (50 ng/ml, PeproTech, Inc., Rocky Hill, NJ, USA) for 7 days. Adherent BMDMs were collected and plated onto 24-well plates at a density of 5 × 10^5^ cells per well. Cells were incubated overnight in serum-free RPMI-1640 medium, and then stimulated by bovine serum albumin (BSA)-conjugated palmitic acid (PA, 500 μM, Cayman chemical, Ann Arbor, MI, USA) for 24 h. BSA-conjugated PA was prepared as previously described (29) with modifications. In another set of experiments, BMDMs were pre-treated with U0126 (10 μM, Promega, Madison, WI, USA) or vehicle (DMSO) for 1 h and then cultured with BSA-conjugated PA for 24 h.

### Adipocyte Differentiation

3T3-L1 preadipocytes (American Type Culture Collection) were cultured in DMEM containing 10% FBS and antibiotics. Differentiation of 3T3-L1 preadipocytes to mature adipocytes was performed, as described (30). Adipocyte differentiation was determined by oil red O staining.

### Short-Interfering (si) RNA and Plasmid Transfection

A total of 1 × 10^6^ adipocytes differentiated from 3T3-L1 preadipocytes at day 8 were transfected with *Spred2*-specific or non-targeting control siRNAs (final concentration 5 pmol; Thermo Scientific, Yokohama, Japan) (17, 31) using a Lipofectamine RNAiMAX (Thermo Scientific) according to the manufacturer's instructions and plated in a 24-well plate. For plasmid transfections, BMDMs were seeded into 24-well plate at a density of 2 × 10^5^ cells per well and incubated overnight in a 5% CO_2_ incubator at 37°C. For each transfection, 0.75 μL Lipofectamine 3000 (Life Technologies, Carlsbad, CA, USA) and 0.5 μg *Spred2* expression plasmid (kindly provided by Dr. Masakiyo Sakaguchi, Okayama university, Japan) were added to 50 μL Opti-MEM (Life Technologies) and incubated for 10 min at room temperature before the mixtures were added to the cells. Cells that overexpressed *Spred2* were grown in RPMI-1640 medium with 10% FBS for 24 h. Cells were then incubated overnight in serum-free RPMI-1640 medium, after which the cells were cultured with BSA-conjugated PA (500 μM) for 24 h.

### Lipolysis Assay and Fatty Acid (FA) Uptake Assay

Epididymal fat pads were minced and digested using type II collagenase (Sigma-Aldrich) dissolved in PBS at 37°C for 20 min on an orbital shaker. The digested tissues were centrifuged at 280 × g for 5 min. The infranatant containing the collagenase solution was removed, and the floating layer of adipocytes was washed three times with PBS. For lipolysis assay, aliquots of isolated adipocytes were incubated in the absence or presence of 100 ng/ml TNFα (R&D systems Inc., Minneapolis, MN, USA) for 3 h. Glycerol content in the infranatant medium was measured using a glycerol colorimetric assay kit (Cayman chemical). To investigate FA uptake, isolated adipocytes were incubated with 20 μM BODIPY FL C16 (Thermo Scientific) in DMEM containing 1%FA-free BSA (Sigma) in the absence or presence of 100 nM insulin (Takara Bio Inc., Shiga, Japan) for 1 h. After the transport period, cells were washed with PBS and then lysed in 0.05N NaOH (32). Fluorescence intensity was measured using FlexStation 3 multi-mode microplate reader (Molecular Devices, LLC. San Jose, CA, USA) at Ex 503 and Em 512 nm, and normalized to intra-droplet fluorescence intensity.

### Histological Analyses

mWAT paraffin sections were stained with hematoxylin and eosin, and the adipocyte size was assessed using the NIH's ImageJ. For immunohistochemistry, sections were incubated with either 10 μg/ml of rat anti-F4/80 antibody (Bio-Rad Laboratories, Inc., Hercules, CA, USA), 2 μg/ml of rabbit anti-iNOS antibody (Abcam plc., Cambridge, MA, USA), or 10 μg/ml of rat anti-CD206 antibody (Bio-Rad) for 90 min, followed by incubation with either Polink-2 Plus HRP Rat-NM with DAB kit or Polink-2 Plus HRP Rabbit with DAB kit (GBI, Inc., Bothell, WA, USA) according to the manufacturer's instructions. Antigen was visualized using 3,3′-diaminobenzidine substrate (Sigma-Aldrich).

### RT-qPCR

Total RNA was extracted from mWAT, adipocytes, or SVF using TRIsure reagent (Bioline Reagents Ltd., London, UK). cDNA was synthesized by the High Capacity cDNA Reverse Transcription Kit (Thermo Scientific) using 500 ng of total RNA. RT-qPCR was performed using StepOne Plus Real-Time PCR system with Taqman PCR master mix (Thermo Scientific). The primers used in this study were: *Spred2* (Mn01223872_g1), *tnfa* (Mm00443258_m1), *mcp-1* (Mm00441242_m1), *adiponectin* (Mm00456425_m1), *leptin* (Mm00434759_m1), *pai1* (Mm00435858_m1), and *rplp0* (Mm00725448_s1) (Thermo Scientific). The expression level of the gene of interest was normalized against *rplp0* and expressed as fold-increases relative to the negative control for each treatment at each time point as previously described (17, 33).

### Western Blotting

Cells were lysed using a lysis buffer (Cell Signaling Technology Inc., Danvers, MA, USA). After centrifugation, clear supernatants were collected and stored at −80°C until further use. The protein concentration in the lysates was measured using protein-dye binding assay (Bio-Rad). Equal amounts (15 μg) of cell lysates were fractionated using sodium dodecyl sulfate–polyacrylamide (SDS) gel electrophoresis (Thermo Scientific), and then proteins were transferred to nitrocellulose membrane. After blocking with TBS-T (Tris-buffered saline containing 0.1% Tween-20) containing 5% BSA, the membranes were incubated with an antibody against either phosphorylated ERK or total ERK (Cell Signaling Technology Inc.) overnight. After washing with TBS-T, membranes were incubated with horseradish peroxidase-conjugated anti-rabbit IgG antibody (Cell Signaling Technology Inc.), and each protein present on the membrane was visualized using an enhanced chemiluminescence system (ImmunoStar LD; Wako Pure Chemical Industries Inc.). Blots were photographed and analyzed using the Image Studio software.

### Enzyme-Linked Immunosorbent Assay (ELISA)

The concentration of TNFα or MCP-1 was measured using a standard sandwich ELISA, as previously described (34, 35). The primary antibody, detection antibody, and recombinant TNFα and MCP-1 were purchased from R&D Systems. The ELISAs used in this study did not cross-react with other known murine cytokines.

### Statistics

All data were expressed as the mean ± SEM. Data were analyzed using the GraphPad Prism software (GraphPad Software, San Diego, CA). Student's *t*-test and analysis of variance (ANOVA) were performed to determine the statistical significance. *P*-values smaller than 0.05 were considered significant.

## Results

### Enhanced Diet-Induced Obesity in Spred2 KO Mice

**Figure 1:**
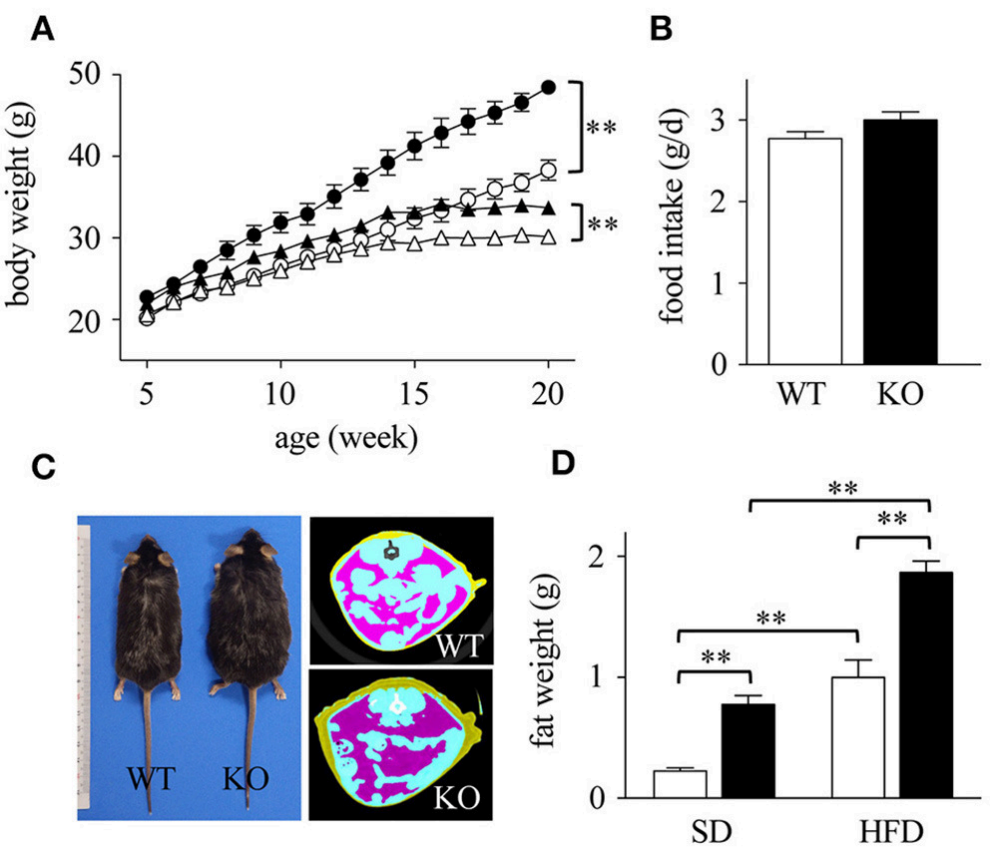
Enhanced diet-induced obesity in Spred2 KO mice. WT and Spred2 KO mice were fed *ad libitum* with either a standard diet (SD) or a high-fat diet (HFD) from 4 to 20 weeks of age. **(A)** Body weight of WT and Spred2 KO mice. Δ, SD-WT; ▴, SD-Spred2KO; ◦, HFD-WT; •, HFD-Spred2 KO. ^**^p < 0.01 (6 mice each group). **(B)** SD-food intake of WT (□) and Spred2 KO (■) mice. Data are shown as average food intake per day from 4 to 6 weeks of age (6 mice each group). **(C)** Representative appearance (left) and computed tomography (CT) image (right; blue, intestine; purple, abdominal adipose tissue) of HFD-fed WT and Spred2 KO mice at the age of 20 weeks. **(D)** The weight of mWATs of SD—or HFD-fed WT (□) or Spred2 KO mice (■). ^**^p < 0.01 (SD: 4 mice each group, HFD: 6 mice each group).

We first compared the weights of WT and Spred2 KO mice fed with SD. Spred2 KO mice exhibited increased body weight compared to WT mice (Figure 1A). Since HFD leads to obesity, particularly in C57BL/6J background (36, 37), we next fed WT and Spred2 KO mice with HFD and examined their weights. As shown in Figure 1A, HFD led to increased body weight in both WT and Spred2 KO mice; however, Spred2 KO mice gained significantly more weight than WT mice (27% heavier) than WT mice after 20 weeks. No difference was found in food consumption between the groups (Figure 1B). There was also no significant difference in the body length between the two strains. CT images demonstrated increased mass of abdominal fat in Spred2 KO mice than in WT mice (Figure 1C). mWATs were subsequently weighed after 16 weeks of SD or HFD. mWATs of Spred2 KO mice were heavier than those of WT mice, when fed with either SD or HFD (Figure 1D). These results clearly indicated that Spred2-deficiency enhanced diet-induced obesity.

### Enhanced Adipocyte Hypertrophy and Dyslipidemia in Spred2 KO Mice

**Figure 2:**
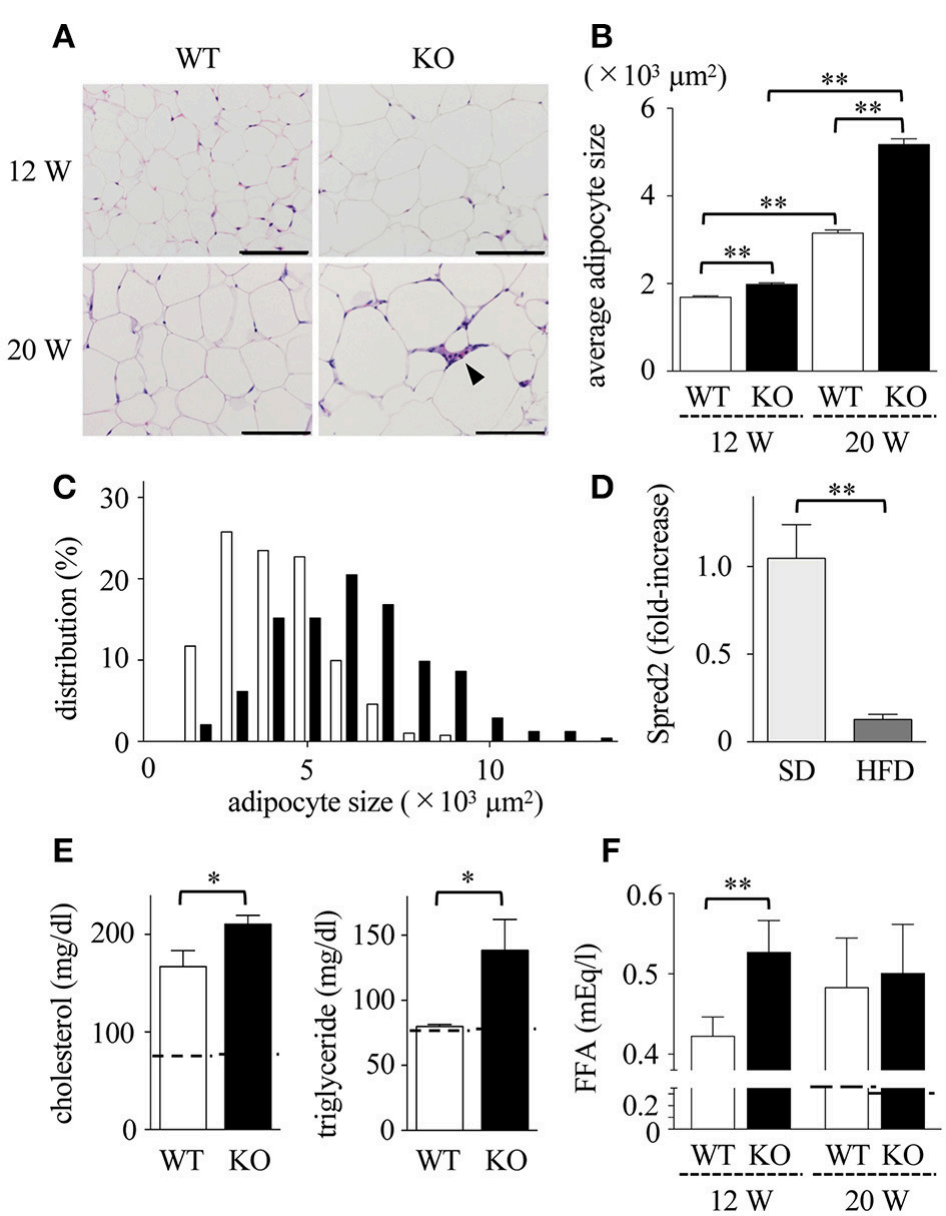
Enhanced adipocyte hypertrophy and dyslipidemia in Spred2 KO mice. WT and Spred2 KO mice were fed with HFD from 4 to 20 weeks of age. Mice were euthanized at 12 weeks or 20 weeks, and mWATs were resected at the time of sacrifice. **(A)** Representative hematoxylin and eosin (H&E) staining sections (scale bar, 100 μm). Crown-like structure is indicated by arrow heads. **(B)** Adipocyte size in mWAT from WT (□) or Spred2 KO (■) was measured. ^**^p < 0.01 (6 mice each group). **(C)** The distribution of adipocyte size at 20 weeks of age. **(D)** mRNA expression of Spred2 in mWATs of WT mice fed with SD or HFD at 20 weeks of age were analyzed by RT-qPCR. The expression levels of Spred2 mRNA were normalized to that of *rplp0*. ^**^p < 0.01 (6 mice each group). The samples harvested at 4 weeks of age were used as baseline controls. **(E)** Serum levels of total cholesterol and triglyceride from WT (□) and Spred2 KO (■) mice fed with HFD at 20 weeks of age were measured. ^*^p < 0.05 (6 mice each group). Dotted line represents data from mice fed with SD from 4 to 20 weeks (mean from 4 mice). **(F)** Serum levels of free fatty acid (FFA) from WT mice (□) and Spred2 KO mice (■) were measured. ^**^p < 0.01 (6 mice each group). Dotted line represents data from mice fed with SD from 4 to 20 weeks (mean from 4 mice).

Obesity is associated with dynamic adipose tissue remodeling characterized by adipocyte hypertrophy (38, 39). As shown in Figures 2A,B, the size of adipocytes in mWAT increased with age (in weeks) in HFD-fed WT and Spred2 KO mice. The size of adipocytes in Spred2 KO-mWAT was significantly larger than that in WT-mWAT, especially after 16 weeks of HFD (Figure 2B). The distribution of adipocyte size shifted toward larger size in Spred2 KO-mWAT after 16 weeks of HFD (Figure 2C).

HFD may affect the level of Spred2 mRNA expression in mWAT. As shown in Figure 2D, Spred2 mRNA expression in WT-mWAT significantly decreased when fed with HFD compared to SD. Next, the serum lipid concentration was measured (Figure 2E). HFD caused an elevation in serum levels of total cholesterol and triglycerides at the age of 20 weeks. FFA levels in HFD-Spred2 KO mice were also significantly higher than those in WT mice after 8 weeks of HFD and remained elevated after 16 weeks of HFD (Figure 2F). Thus, over-nutrition caused by HFD exacerbated dyslipidemia in Spred2 KO mice.

### Exacerbated Adipose Tissue Inflammation in Spred2 KO Mice

**Figure 3:**
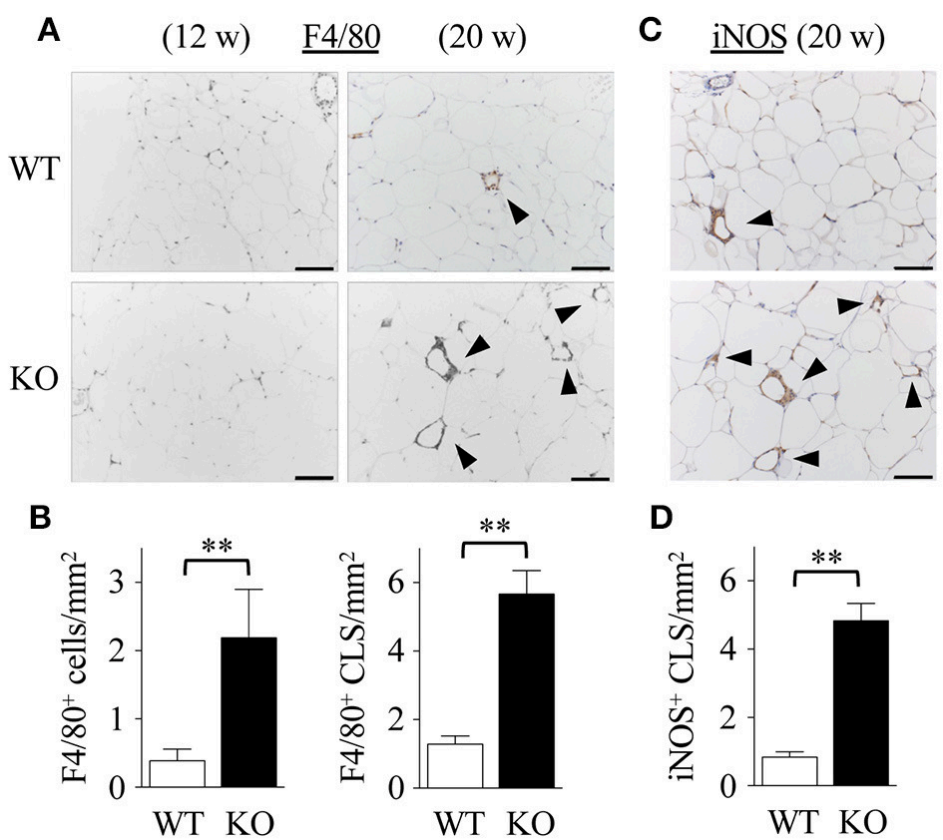
Exacerbated adipose tissue inflammation in Spred2 KO mice. WT and Spred2 KO mice were fed with HFD from 4 to 20 weeks of age. Mice were euthanized at 12 weeks or 20 weeks, and mWATs were resected at the time of sacrifice. **(A)** Macrophages were stained with anti-F4/80 Ab (original scale bar, 100 μm). Representative results from 6 mice are shown. Crown-like structure (CLS) is indicated by arrow heads. **(B)** The number of F4/80^+^ cells and F4/80^+^ CLS was counted at 20 weeks of age. □, WT; ■, Spred2 KO. ^**^p < 0.01 (6 mice each group). **(C)** Macrophages were stained with anti-iNOS Ab (original scale bar, 100 μm). Representative results from six mice are shown. **(D)** The number of iNOS^+^ CLS (arrow heads) was counted at 20 weeks of age. □, WT; ■, Spred2 KO. ^**^p < 0.01 (6 mice each group).

Dyslipidemia may lead to low-grade inflammation, characterized by macrophage infiltration in adipose tissue (40). Immunohistochemical analyses revealed that the number of F4/80 positive macrophages (Figure 3A) in Spred2 KO-mWAT was significantly higher than that in WT-mWAT (Figure 3B, left). Crown-like structures, composed of macrophages surrounding dead or dying adipocytes (Figure 3A) (41), were significantly increased in Spred2 KO-mWAT (Figure 3B, right). Macrophages migrating in mWAT are known to have M1 phenotype (pro-inflammatory macrophages) (42). Consequently, we performed immunostaining using antibodies against either inducible nitric oxide synthase (iNOS) or CD206, known representative markers for M1 or M2, respectively, (43). The number of iNOS^+^ macrophages (Figure 3C) was significantly higher in Spred2 KO-mWAT than that in WT-mWAT (Figure 3D), whereas there were only a few CD206^+^ macrophages in both groups and M1:total macrophage ratio was similar between both groups (data not shown). These results suggested that Spred2 KO mice exhibited exacerbated infiltration of pro-inflammatory macrophages.

### Impaired Insulin Resistance and Fatty Liver Disease in Spred2 KO Mice

**Figure 4:**
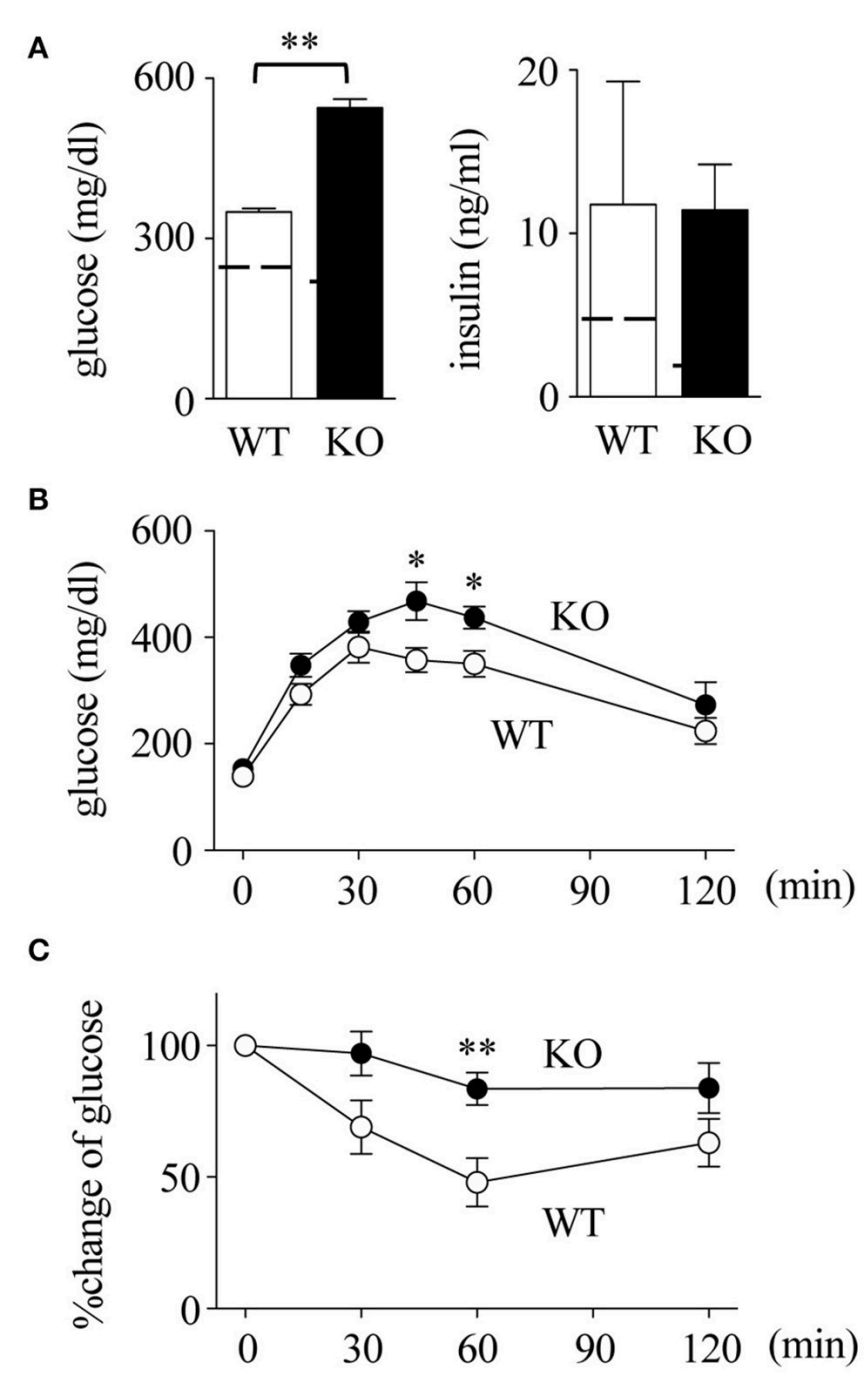
Impaired insulin resistance in Spred2 KO mice. WT and Spred2 KO mice were fed with HFD from 4 to 20 weeks of age. **(A)** Mice were euthanized at 20 weeks, and serum levels of glucose and insulin were measured. ^**^p < 0.01 (6 mice each group). Dotted line represents data from mice fed with SD from 4 to 20 weeks (mean from 4 mice). **(B,C)**. Blood glucose levels after intraperitoneal injection of **(B)** glucose (1 g/kg body weight) or **(C)** insulin (1 U/kg body weight) were measured at 20 weeks of age. Percent changes are shown in **(C)**. ◦, WT; •, Spred2 KO. ^*^p < 0.05, ^**^p < 0.01 (6 mice each group).

Obesity-induced adipose tissue inflammation leads to insulin resistance (4, 44, 45). As shown in Figure 4A, serum levels of glucose in Spred2 KO mice were significantly higher than those in WT mice, although the insulin levels were similar between the groups. Consequently, *in vivo* metabolic tests were performed, which showed that both glucose intolerance and insulin resistance were aggravated in Spred2 KO mice when compared with WT mice (Figures 4B,C). Thus, Spred2 KO mice showed impaired insulin resistance.

**Figure 5:**
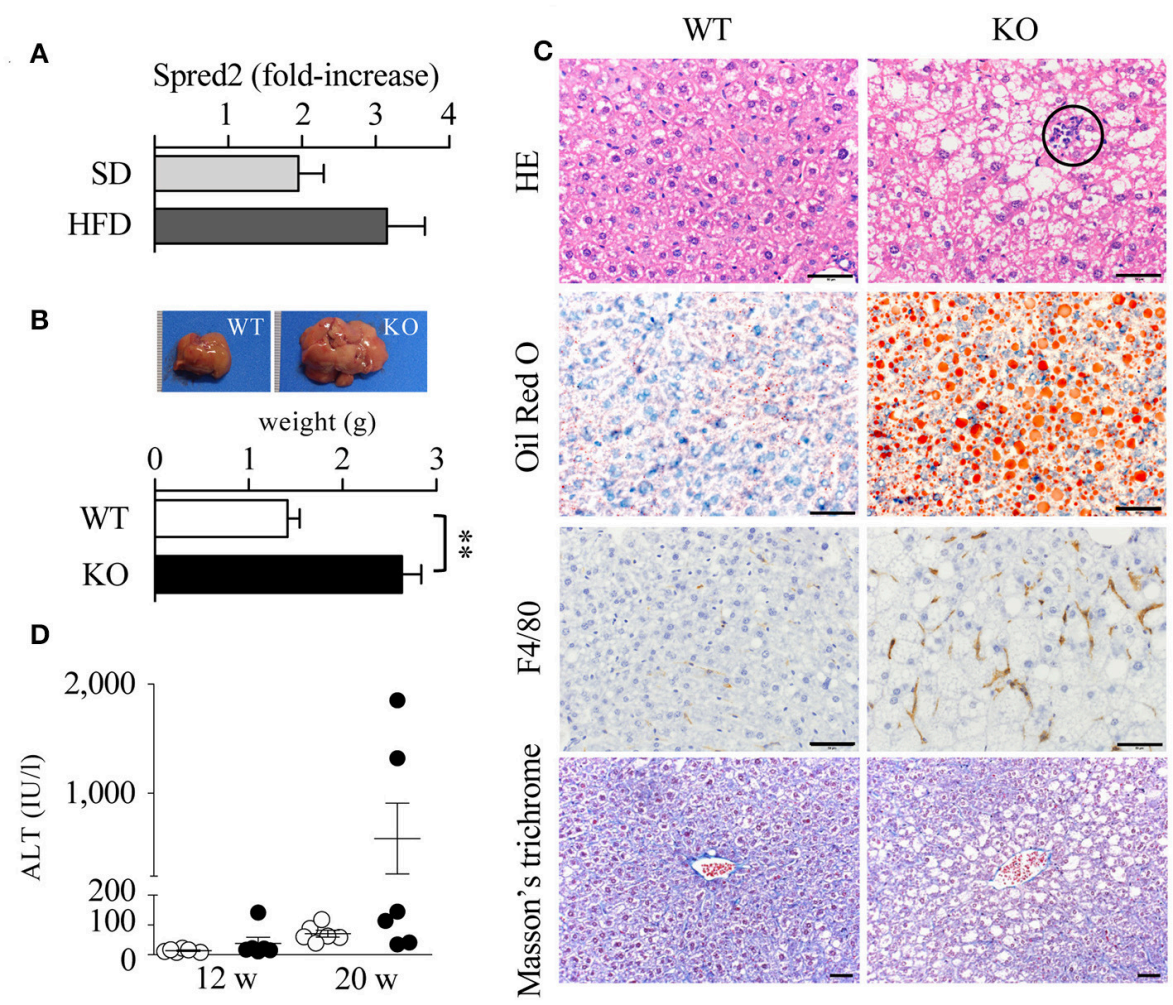
Exacerbated fatty liver disease in Spred2 KO mice. **(A)** WT mice were fed with either SD or HFD (6 mice each group) from 4 to 20 weeks of age. Livers were resected at 20 weeks of age and Spred2 mRNA expression was quantitated. The expression levels of Spred2 mRNA were normalized to that of *rplp0*. The samples harvested at 4 weeks of age were used as baseline controls. **(B–D)** WT and Spred2 KO mice were fed with HFD from 4 to 20 weeks of age. **(B)** The livers were resected at 20 weeks of age. Upper panel, a representative gross appearance of the liver from WT or Spred2 KO mice was shown. Lower panel, the livers were weighed. ^**^p < 0.01 (6 mice each group). **(C)** The liver tissues at 20 weeks of age were stained with H&E, Oil Red O, for F4/80 (immunostaining), or Masson's trichrome (scale bar, 50 μm). Circle area; neutrophil infiltration. Representative photos from each group were shown (6 mice each group). **(D)** Serum ALT levels were measured at 12 and 20 weeks. ◦WT, •KO (6 mice each group, each time point).

The liver plays a principal role in lipid metabolic pathways. Increased serum lipid levels and impaired insulin resistance may cause non-alcoholic fatty liver disease (NAFLD)/non-alcoholic steatohepatitis (NASH) (46) in Spred2 KO mice. The expression of Spred2 in WT-livers tended to increase when mice were fed with HFD (*p* = 0.12, Figure 5A). The data in Figure 5B demonstrated that HFD-Spred2 KO-livers were heavier than WT-livers. Histologically, in comparison with WT-livers, Spred2 KO-livers revealed moderate vesicular steatosis, and influx of neutrophils and F4/80-positive macrophages. Perivenular/pericellular fibrosis was not evident by Masson's trichrome staining (Figure 5C). The serum levels of ALT, a marker of liver injury, tended to increase in Spred2 KO mice as compared with WT mice (*p* = 0.14, after 16 weeks of HFD, Figure 5D). These data indicated that Spred2 KO mice developed NAFLD/NASH.

### Enhanced Cytokine Response in Spred2 KO Mice

**Figure 6:**
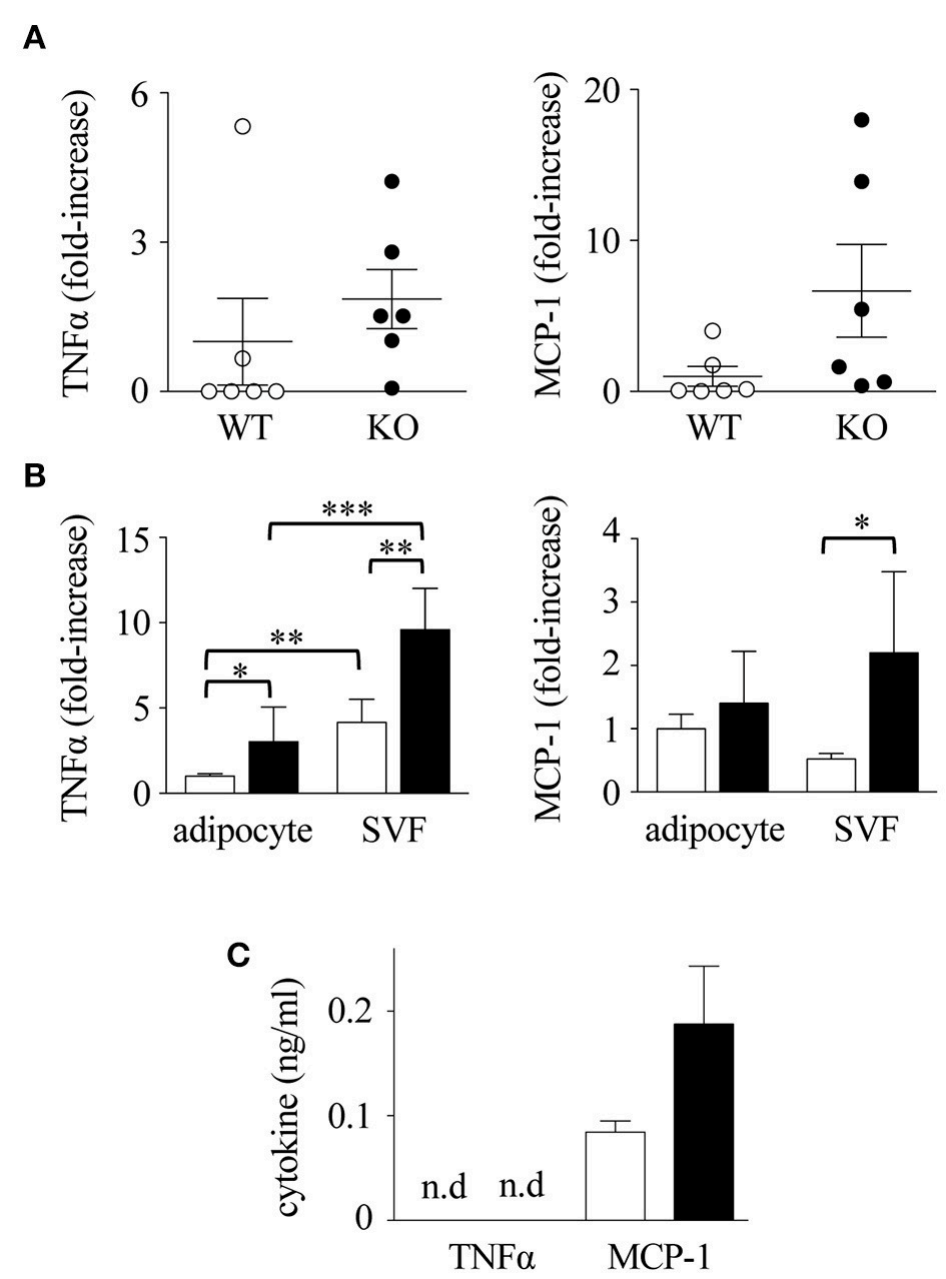
Enhanced cytokine response in Spred2 KO mice. WT and Spred2 KO mice were fed with HFD from 4 to 20 weeks of age, and euthanized at 20 weeks of age. **(A)** mWATs were resected and mRNA expression of TNFα (left) and MCP-1 (right) was quantitated. The expression levels of Spred2 mRNA were normalized to that of *rplp0*. ◦, WT; •, Spred2 KO. **(B)**. mWATs were divided into adipocytes and stromal vascular fraction (SVF), and mRNA expression of TNFα (left) and MCP-1 (right) were quantitated. The expression levels of each mRNA were normalized to that of *rplp0*. □, WT; ■, Spred2 KO. ^*^p < 0.05, ^**^p < 0.01, ^***^p < 0.001 (6 mice each group). **(C)** Serum levels of TNFα and MCP-1 (8 mice each group) were measured. □, WT; ■, Spred2 KO.

Macrophage infiltration in mWAT can be induced by cytokines, among which TNFα and MCP-1 appear to be crucial (47, 48). To examine the cytokine response, we examined the expression of TNFα and MCP-1 mRNA in mWATs obtained from mice fed with HFD. After 16 weeks of HFD, mRNA levels of TNFα and MCP-1 in Spred2 KO-mWAT tended to be higher than those in WT-mWAT (Figure 6A), although the data was not statistically significant (TNFα; *p* = 0.43, MCP-1; *p* = 0.10). Next, mWATs were divided into adipocytes and stromal vascular fraction (SVF) that contained preadipocytes, endothelial cells, and macrophages (49), and the cytokine responses of both fractions were examined. As shown in Figure 6B, expression levels of TNFα and MCP-1 were 2.2-fold and 3.1-fold higher in Spred2 KO-SVF, respectively, than those in WT-SVF. The expression level of TNFα was higher in Spred2 KO-adipocytes than that in WT-adipocytes, but its expression level was lower than that in the SVF. No difference was found in the expression levels of adipocyte-MCP-1 between the mouse groups (Figure 6B). Interestingly, expression of circulating MCP-1/CCL2 tended to be higher in Spred2 KO mice than that in WT mice (*p* = 0.09), despite no detection of TNFα in either group (Figure 6C). The levels of other adipokines, including adiponectin, leptin, and plasminogen activator inhibitor-1 (PAI-1) in the adipocytes were similar between the groups, although, a decreasing trend was observed in the levels of adiponectin (*p* = 0.14, 6 mice each, Supplementary Data 1). These results suggested that SVF preferentially plays an important role in the regulation of adipose tissue inflammation by generating TNFα and MCP-1.

### Cytokine Production and Lipid Metabolism in Spred2 Deficiency

Spred2 in adipocytes may regulate cytokine production and lipid metabolism. For cytokine production, we knocked down Spred2 expression (~80% reduction) in adipocytes differentiated from 3T3-L1 preadipocytes using short interfering (si)RNA and the cells were stimulated with TNFα (10 ng/ml) for 24 h. Spred2 knockdown did not alter the expression levels of *de novo* TNFα and MCP-1 as compared to the controls (data not shown), suggesting that the role of Spred2 in adipocyte cytokine production is minimal. For lipid metabolism, we compared glycerol release and fatty acid uptake between primary WT-and Spred2 KO-adipocytes. No difference was found in the glycerol release (WT vs. Spred2 KO, 3.12 ± 0.07 vs. 3.42 ± 0.24 mg/l/10^6^ cells, *n* = 4 each, not significant) and fatty acid uptake (WT vs. Spred2 KO, 1.20 ± 0.20 vs. 1.32 ± 0.26/normalized fluorescence, *n* = 4 each, not significant) between the groups after the stimulation with TNFα and insulin, respectively. To examine the involvement of Spred2 in adipocyte hypertrophy, we knocked down Spred2 expression (~80% reduction) in adipocytes differentiated from 3T3-L1 preadipocytes and cultured for 21 days. After oil red O staining, adipocyte hypertrophy was quantified by extracted oil red O level. Spred2 knockdown did not alter the amount of lipid contents (control vs. Spred2 knockdown, 0.52 ± 0.02 vs. 0.49 ± 0.07/absorbance at 490 nm, *n* = 3 each, not significant). These data suggest that Spred2 in adipocytes does not play a significant role in cytokine production and lipid metabolism.

### Involvement of ERK/MAPK in Macrophage Cytokine Response

**Figure 7:**
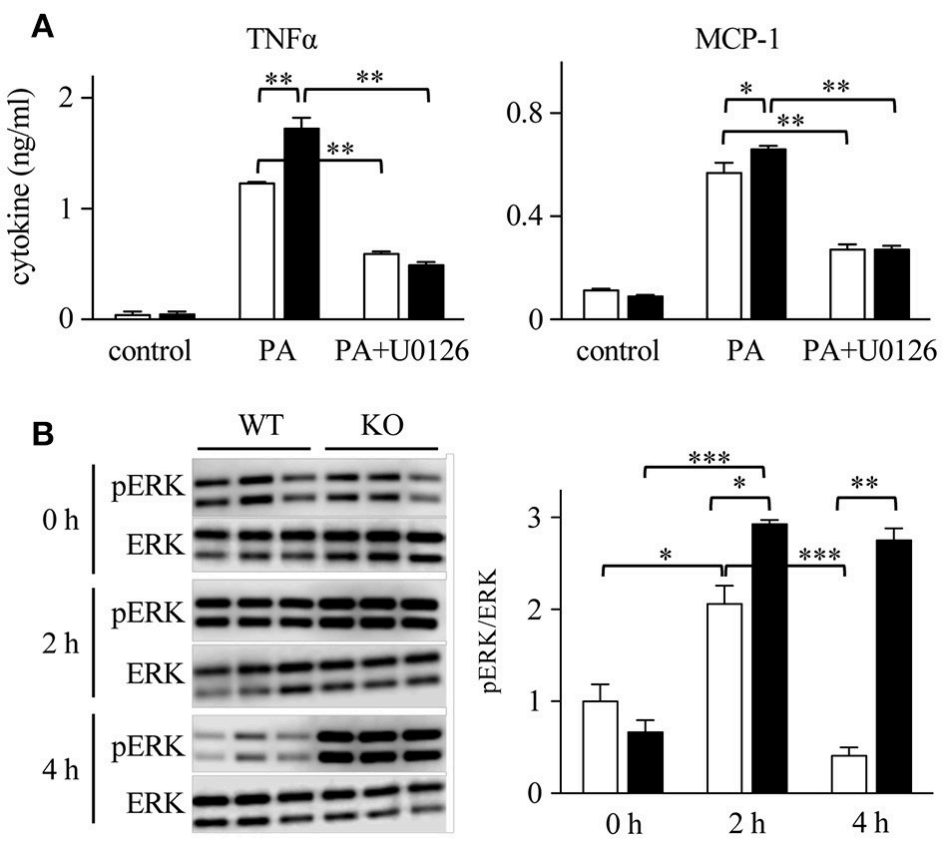
The involvement of ERK/MAPK in macrophage cytokine response. BM cells were harvested from WT and Spred2 KO mice and differentiated into BMDM. **(A)** Macrophages from WT (□) or Spred2 KO (■) mice were stimulated with palmitate acid (PA) (500 nM) with or without U0126 (10 μM) treatment for 24 h. Cytokine levels in the culture supernatants were measured by ELISA. ^*^p < 0.05, ^**^p < 0.01 (3 samples each group). **(B)** Macrophages from WT (□) or Spred2 KO (■) mice were stimulated with PA for 2 and 4 h. Left, immunoblot data from three independent experiments. Right, the density of each band was digitalized and semi-quantitated. ^*^p < 0.05, ^**^p < 0.01, ^***^p < 0.001 (3 samples each group, each time point).

We hypothesized that macrophages, a major structural component of SVF, are the main cells involved in the production of cytokines. To confirm the role of macrophages in mWAT, we employed bone marrow-derived macrophages (BMDMs), as majority of adipose tissue macrophages are derived from the bone marrow (50). Upon stimulation of macrophages with SFA (palmitic acid; PA), Spred2 KO-macrophages produced significantly higher levels of TNFα and MCP-1 than WT-BMDMs (Figure 7A). However, pretreatment with U0126, a selective MEK/ERK inhibitor, significantly reduced the production of TNFα and MCP-1 in both WT-and Spred2 KO-macrophages (Figure 7A), suggesting that the cytokine response was dependent on the ERK/MAPK pathway. In addition, the phosphorylation of ERK was significantly enhanced in Spred2 KO-macrophages compared to WT-macrophages after PA-stimulation (Figure 7B, left). The phosphorylation of ERK in WT-macrophages returned to the basal level by 4 h, whereas levels of ERK phosphorylation remained elevated in Spred2 KO-macrophages (Figure 7B, right). Thus, macrophages appear to be responsible for the enhanced production of TNFα and MCP-1 in absence of Spred2.

### *Spred2* Overexpression Reduced Cytokine Responses in Macrophages

**Figure 8:**
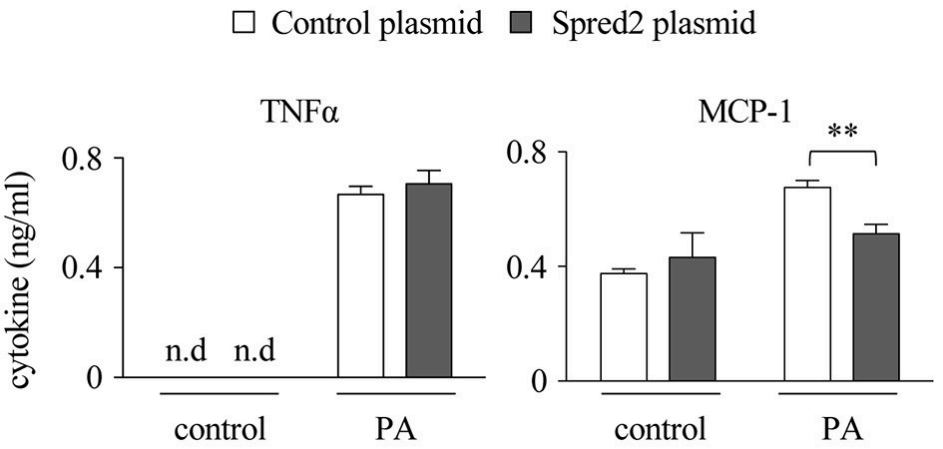
*Spred2* overexpression reduced cytokine responses in macrophages. *Spred2* was overexpressed by transfecting macrophages with mixtures of Lipofectamine 3000 and *Spred2* overexpression plasmid and control plasmid for 24 h, after which the cells were stimulated with PA (500 μM) for 24 h. Cytokine levels in the culture supernatants were measured by ELISA. ^**^p < 0.01 (4 samples each group).

The above data raised the question of whether greater amounts of Spred2 in a cell could reduce cytokine responses. To address this, we overexpressed *Spred2* in WT-macrophages using an overexpression plasmid that increased *Spred2* expression by 90-fold, as determined by Taqman RT-qPCR (data not shown). Although no difference was found in the level of TNFα, *Spred2* overexpression significantly reduced MCP-1 level after PA stimulation compared with the control cells (Figure 8). Thus, Spred2 overexpression effectively reduced PA-induced MCP-1, but not TNFα response. Endogenous Spred2 in macrophages may be sufficient to down-regulate TNFα response.

## Discussion

Previous studies have shown that the MAPK pathway, especially the ERK/MAPK pathway plays an important role in obesity and obesity-associated insulin resistance (13, 51). Genome-wide association studies have indicated that a genetic variation near the Sprouty homolog 2 (*SPRY2*) gene, an inhibitor of the Ras/Raf/ERK/MAPK pathway (52), is associated with body fat percentage in human (53, 54). In the present study, we explored the role of Spred2 in obesity and obesity-associated systemic responses, and found that Spred2 KO mice developed exacerbated obesity, hyperlipidemia, hyperglycemia, insulin resistance, and fatty liver upon feeding with HFD, reflecting a severe state of metabolic syndrome. Thus, for the first time, we identified Spred2 as an important molecule that regulates obesity and obesity-associated metabolic abnormalities.

It has been shown that ERK1 KO mice have decreased adiposity and fewer adipocytes than WT mice and are resistant to obesity induced by HFD (14). The ERK/MAPK pathway is crucial for the differentiation of 3T3-L1 cells into adipocytes (55). Inhibition of the ERK/MAPK pathway in 3T3-L1 adipocytes by MEK inhibitors ameliorated the adipokine expression and suppressed the lipolytic activity (56). TNFα-induced lipolysis was inhibited by U0126 or PD98059, a selective MEK/ERK inhibitors, in 3T3-L1 adipocytes (57). Thus, ERK/MAPK appears to be involved in adipocyte hypertrophy and lipolysis. We demonstrated in this study that the Spred2 KO mice exhibited an exacerbated level of adipocyte hypertrophy and hyperlipidemia (elevated levels of cholesterol, triglyceride, and FFA), suggesting that Spred2 negatively regulates adipocyte hypertrophy and hyperlipidemia.

Interestingly, Spred2 expression in WT-mWATs was dramatically reduced by HFD, although the expression was increased in the liver. When BMDMs were stimulated with PA, Spred2 expression was significantly reduced whereas the expression was increased when adipocytes differentiated from 3T3-L1 preadipocytes were stimulated with TNFα (Supplementary Data 2). These data represent the unique behavior of Spred2 expression in different tissues and cell-types. It remains unclear how HFD decreases the Spred2 expression in WT-mWAT; however, HFD may drive epigenetic changes that result in Spred2 gene regulation. Further studies will be necessary to identify the precise molecular mechanisms involved.

Various inflammatory mediators are involved in adipose tissue inflammation. Among them, a paracrine loop linking fatty acids, TNFα, and MCP-1 establish a vicious cycle between adipocytes and macrophages that aggravates inflammation in the adipose tissues (47). TNFα and MCP-1 are adipokines (48) and are also produced from macrophages. Fatty acids released from the hypertrophied adipocytes appear to preferentially stimulate macrophages to produce TNFα and MCP-1, as levels of TNFα and MCP-1 in Spred2 KO-SVF were significantly higher compared to adipose tissues. In addition, Spred2 KO-macrophages expressed increased levels of TNFα and MCP-1 upon stimulation with SFA (PA). Spred2 knockdown did not alter TNFα and MCP-1 levels in 3T3-L1 adipocytes. Thus, our data suggested the involvement of macrophages rather than adipocytes in enhanced TNFα and MCP-1 expression in Spred2 KO mice.

Spred2 deficiency may have a more direct effect on adipocyte fat accumulation and/or lipolysis. However, lipid metabolism, as assessed by glycerol release and fatty acid uptake, was unchanged between WT-and Spred2 KO-adipocytes, and Spred2 knockdown failed to alter 3T3-L1 adipocyte hypertrophy. Cytokine, especially TNFα, is known to release FFA from adipocytes. Although Spred2 KO mice have increased fat mass under SD treatment, there was no apparent adipose tissue inflammation and dyslipidemia (data not shown). It is possible that augmented inflammation in Spred2 deficiency under HFD affect adipose tissue pathology via upregulated cytokine response by macrophages. Additional study is required to obtain more definitive answers.

To determine the contribution of Spred2 in macrophages, we employed bone marrow chimeric mice, and examined their weights and performed glucose and insulin tolerance tests after being fed on HFD; however, no significant differences were found between the groups (Supplementary Data 3), likely due to the adipose tissue alteration to radiation exposure (58). Irradiation could alter the properties of adipocytes. Conditional macrophage—or adipocyte-specific Spred2 may reveal such mechanisms in detail. Macrophage response could be mediated through TLR4 (6, 20). The TLR4 ligand LPS stimulated bone marrow-derived macrophages to produce TNFα and MCP-1 (17). We recently showed that macrophage cytokine response to LPS was partially blocked by U0126 (24), suggesting that Spred2 may be involved in the regulation of the MAPK pathway downstream of TLR4. It is also possible that Spred2 may suppress TLR signaling by other unidentified mechanisms.

Spred2 deficiency resulted in fatty liver hepatitis without liver fibrosis. Previous studies have revealed that HFD-fed mice developed liver fibrosis after 9.5 months (59) or 1 year (60), suggesting a possibility that Spred2 KO mice may develop NASH with liver fibrosis after being fed for a long period of HFD. Further studies are necessary to investigate the contribution of Spred2 in development of NAFLD/NASH, and subsequent hepatocellular carcinoma.

In conclusion, we showed that Spred2-deficiency promotes obesity, adipose tissue inflammation, and insulin resistance, possibly via enhanced adipose tissue inflammation caused by enhanced macrophage activation. A fascinating question is whether the abnormal metabolic status induced by high calorie diet can be treated by Spred2 supplementation *in vivo*. Spred2 may act as a novel therapeutic tool to treat the progression of pathophysiology associated with metabolic syndrome.

## Author Contributions

TaO, TY, and AM planned experiments and wrote the main manuscript text. TaO, RM, and KU performed experiments, and discussed the experimental findings and interpretation of results. ToO and MF discussed the experimental findings and interpretation of results. All authors reviewed the manuscript.

### Conflict of Interest Statement

The authors declare that the research was conducted in the absence of any commercial or financial relationships that could be construed as a potential conflict of interest.
