# Supplementary figures and images for: Spred2 Regulates High Fat Diet-Induced Adipose Tissue Inflammation, and Metabolic Abnormalities in Mice
Source: Front Immunol. 2019 Jan 22;10:17. doi: 10.3389/fimmu.2019.00017 (PMC6349710; doi:10.3389/fimmu.2019.00017)

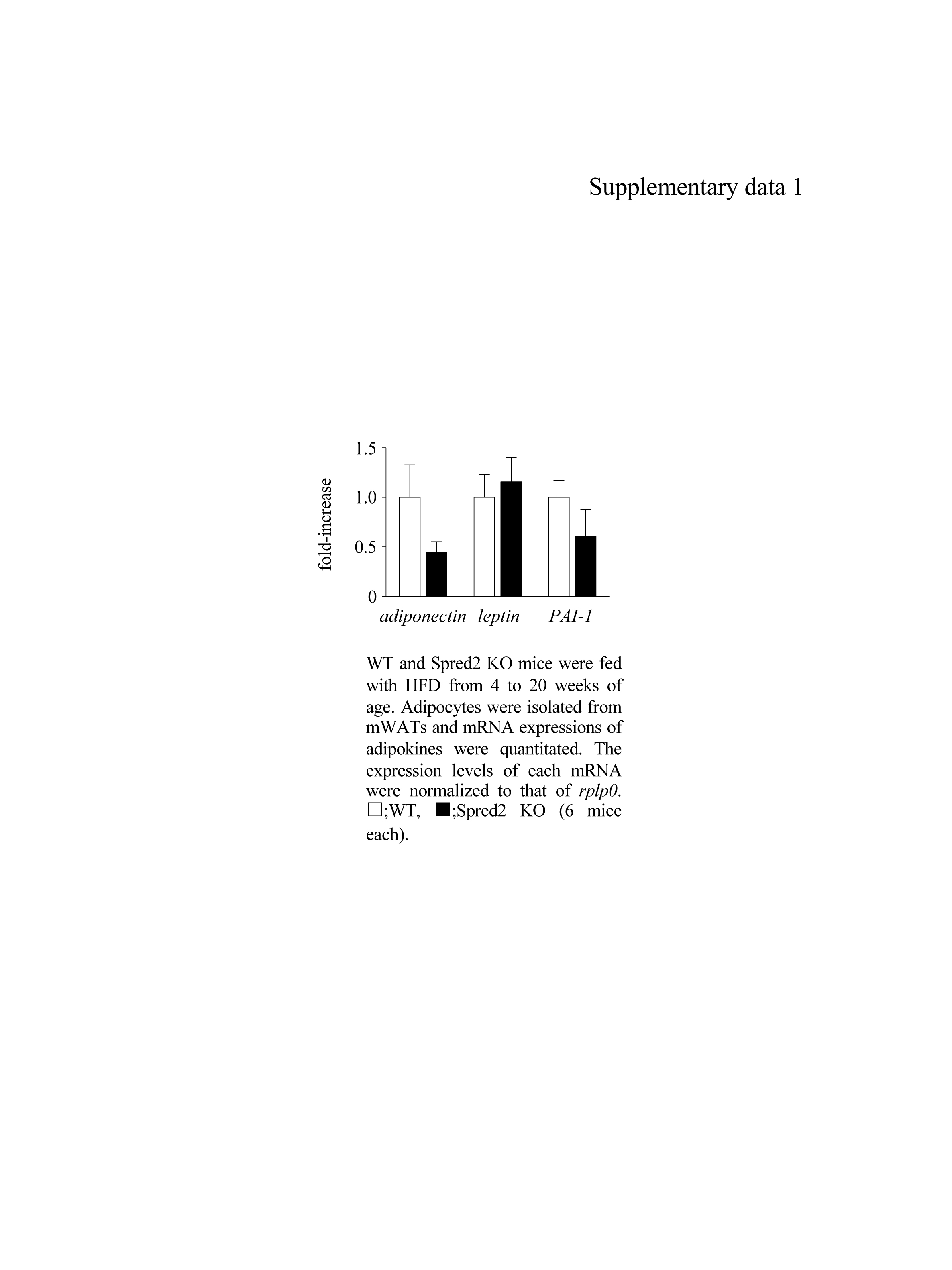

Supplement: Supplementary file 1 [file Image_1.JPEG]

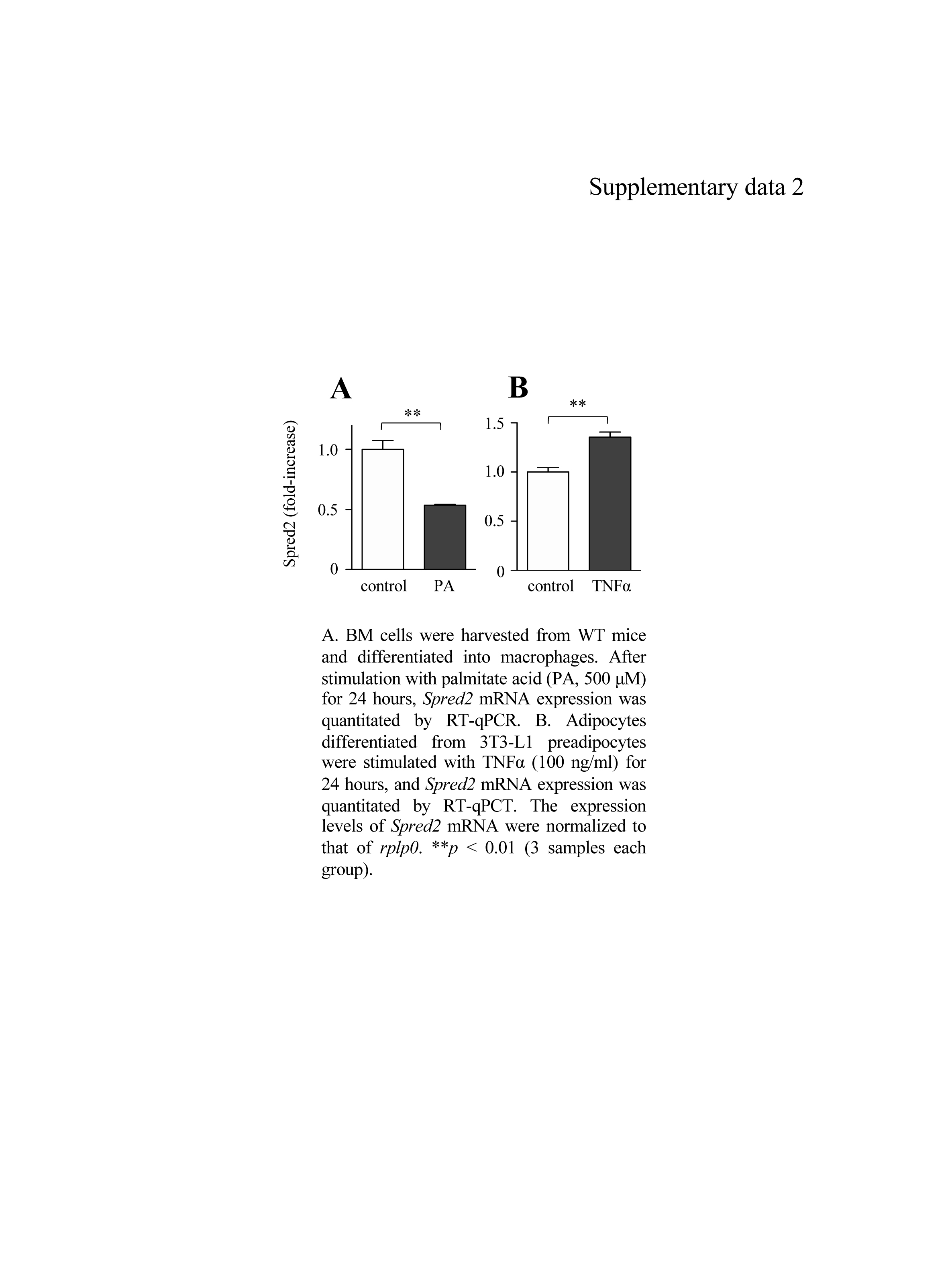

Supplement: Supplementary file 2 [file Image_2.JPEG]

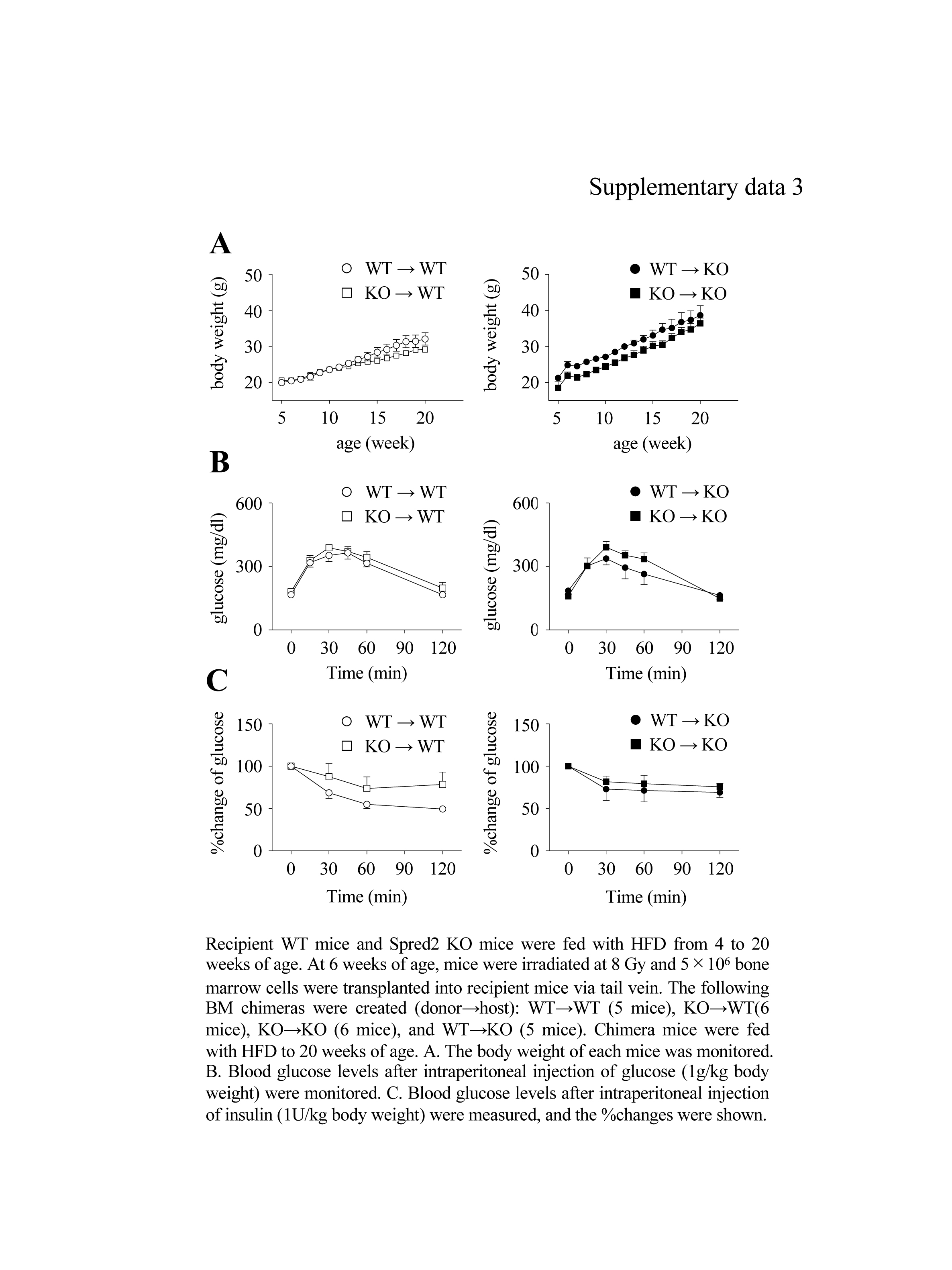

Supplement: Supplementary file 3 [file Image_3.JPEG]
